# Characteristics and Prognostic Relevance of Ventricular Arrhythmia in Patients with Myocarditis

**DOI:** 10.3390/jcdd9080243

**Published:** 2022-07-29

**Authors:** Ann-Kathrin Kahle, Rebekka Güde, Jana M. Schwarzl, Paula Münkler, Ruken Ö. Akbulak, Charlotte Jahnke, Sebastian Bohnen, Tilman Würger, Michael Schwarzl, Stephan Willems, Ulf K. Radunski, Christian Meyer

**Affiliations:** 1 Division of Cardiology, Angiology, Intensive Care Medicine, EVK Düsseldorf, cNEP, cardiac Neuro- and Electrophysiology Research Consortium, Kirchfeldstrasse 40, 40217 Düsseldorf, Germany; ann-kathrin-kahle@gmx.de; 2 Department of Cardiology, Pulmonology and Vascular Medicine, Medical Faculty, University Hospital Düsseldorf, Moorenstrasse 5, 40225 Düsseldorf, Germany; 3 Institute of Neural and Sensory Physiology, cNEP, cardiac Neuro- and Electrophysiology Research Consortium, Medical Faculty, Heinrich Heine University Düsseldorf, Universitätsstrasse 1, 40225 Düsseldorf, Germany; 4 Clinic for Cardiology, University Heart & Vascular Center, University Hospital Hamburg-Eppendorf, Martinistrasse 52, 20246 Hamburg, Germany; rebekka.guede@gmail.com (R.G.); praxis@kardio-innsbruck.at (J.M.S.); p.muenkler@uke.de (P.M.); c.jahnke@uke.de (C.J.); t.wuerger@uke.de (T.W.); michael.schwarzl@kh-schwaz.at (M.S.); 5 DZHK (German Centre for Cardiovascular Research), Partner Site Hamburg/Kiel/Lübeck, Potsdamer Strasse 58, 10785 Berlin, Germany; s.willems@asklepios.com; 6 Department of Cardiology and Internal Intensive Care Medicine, Asklepios Hospital St. Georg, Lohmühlenstrasse 5, 20099 Hamburg, Germany; r.akbulak@asklepios.com (R.Ö.A.); s.bohnen@asklepios.com (S.B.); 7 Department of Cardiology, Regio Clinics, Agnes-Karll-Allee 17, 25337 Elmshorn, Germany; u.radunski@gmail.com

**Keywords:** cardiac magnetic resonance imaging, catheter ablation, myocarditis, premature ventricular complexes, ventricular arrhythmia, ventricular fibrillation, ventricular tachycardia

## Abstract

Myocarditis is characterized by various clinical manifestations, with ventricular arrhythmia (VA) as a frequent symptom at initial presentation. Here, we investigated characteristics and prognostic relevance of VA in patients with myocarditis. The study population consisted of 76 patients with myocarditis, verified by biopsy and/or cardiac magnetic resonance (CMR) imaging, including 38 consecutive patients with VA (45 ± 3 years, 68% male) vs. 38 patients without VA (NVA) (38 ± 2 years, 84% male) serving as a control group. VA was monomorphic ventricular tachycardia in 55% of patients, premature ventricular complexes in 50% and ventricular fibrillation in 29%. The left ventricular ejection fraction at baseline was 47 ± 2% vs. 40 ± 3% in VA vs. NVA patients (*p* = 0.069). CMR showed late gadolinium enhancement more often in VA patients (94% vs. 69%; *p* = 0.016), incorporating 17.6 ± 1.8% vs. 8.2 ± 1.3% of myocardial mass (*p* < 0.001). Radiofrequency catheter ablation for VA was initially performed in nine (24%) patients, of whom five remained free from any recurrence over 24 ± 3 months. Taken together, in patients with myocarditis, reduced left ventricular ejection fraction does not predict VA occurrence but CMR shows late gadolinium enhancement more frequently and to a larger extent in VA than in NVA patients, potentially guiding catheter ablation as a reasonable treatment of VA in this population.

## 1. Introduction

Myocarditis shows a great variability of clinical manifestations, with ventricular arrhythmia (VA) as a common symptom at initial presentation [1,2]. Accordingly, disease course of myocarditis varies considerably, and predictors of poor outcome are frequently discussed. In this context, left ventricular ejection fraction (LVEF) at first diagnosis, as well as at reevaluation after 6 months, has been correlated with long-term outcome [3]. Whereas progression to dilated cardiomyopathy (DCM) has been found in up to 30% of cases, other patients present with partial or full recovery. However, late disease relapses, many years after the first episode, have been reported [4]. VA is often transient in the acute setting but can also persist and potentially cause sudden cardiac death [5]. Registry data suggest increased incidence of VA and mortality after diagnosis of myocarditis, irrespective of the development of DCM with impaired LVEF [6]. So far, the characteristics and prognostic relevance of VA at initial presentation concerning disease progression and mortality are unknown. Here, we aimed to investigate our cohort of patients diagnosed with myocarditis with regards to (1) characteristics of VA, (2) differences in cardiac magnetic resonance (CMR) imaging compared to myocarditis patients without VA (NVA) and (3) the prognostic relevance of VA.

## 2. Materials and Methods

### 2.1. Study Population

The study population consisted of 76 patients with myocarditis, verified by endomyocardial biopsy (EMB) and/or CMR, including 38 consecutive patients presenting with VA. For comparison, 38 NVA patients served as a control group.

All patients underwent physical examination, 12-channel electrocardiogram and transthoracic echocardiography. Preserved LVEF was defined as ≥54%, severely impaired LVEF as <30%. Laboratory tests included high-sensitivity cardiac troponin T, C-reactive protein and N-terminal prohormone of brain natriuretic peptide at initial presentation. Coronary angiography to exclude relevant coronary artery disease was performed in all patients with cardiovascular risk factors or >35 years. EMB was conducted after written informed consent by venous access via right internal jugular or femoral approach with acquisition of a minimum of three samples from the right ventricular septum. Diagnosis of myocarditis was made in accordance with the Dallas Criteria and included immunological and immunohistochemical findings as well as viral genome analysis [5,7,8,9].

### 2.2. Ventricular Arrhythmia Characteristics

VA was diagnosed by resting 12-channel electrocardiogram and, whenever available, Holter monitor and device interrogation. Treatment included beta-receptor blockers and antiarrhythmic drugs, depending on clinical presentation. Implantation of a cardioverter-defibrillator (ICD) or prescription of a wearable cardioverter-defibrillator (WCD) was performed according to the guidelines [5]. In case of recurrent VA, radiofrequency catheter ablation (RFCA) was considered and performed, as previously described [10,11,12].

### 2.3. Cardiac Magnetic Resonance Imaging

Diagnosis of myocarditis was made in accordance with the Lake Louise Criteria [13]. CMR was conducted on a 1.5 Tesla scanner (Achieva, Philips Medical Systems, Best, The Netherlands), with a protocol consisting of conventional cine- and edema-sensitive-imaging as well as early myocardial enhancement and late gadolinium enhancement (LGE) CMR on three representative short-axis positions. Left ventricular function and volumes were assessed on a cine-CMR short axis stack using a steady-state free precession sequence. For edema-sensitive T2-weighted imaging, a fat-suppressed black blood triple inversion recovery turbo-spin-echo sequence was used on an end-diastolic left ventricular short axis [14]. For assessment of early gadolinium enhancement, pre- and post-contrast end-diastolic T1-weighted spin-echo images were used [15]. Phase-sensitive inversion recovery LGE imaging was performed after injection of a bolus of 0.075 mmol/kg gadobenate dimeglumine at a rate of 2.5 mL/s [14]. 

For quantification of myocardial injury, a standard threshold technique relative to apparently normal myocardium was used, employing the Circle cvi42^®^ software (Circle Cardiovascular Imaging Inc.; Calgary, AB, Canada) on LGE images: a region of interest was drawn in apparently normal myocardium without LGE and propagated to corresponding LGE images [14]. Myocardium with a mean signal intensity ≥ 3 standard deviations above apparently normal myocardium was defined as injured [16]. LGE amount was calculated as the percentage of pixels with injured myocardium [14]. Assessment of LGE localization was based on the 17-segment model of the American Heart Association, as previously introduced [17].

### 2.4. Treatment and Follow-Up

Immunosuppressive therapy was initiated at the discretion of the treating physician [3]. In the presence of heart failure, medical and device treatment according to the current guidelines was initiated [5,18]. Follow-up visits included physical examination, 12-channel electrocardiogram, transthoracic echocardiography and rhythm-monitoring or device interrogation, if applicable. Visit intervals were determined according to the individual clinical course. In case of disease progression to manifest DCM with reduced LVEF, patients were seen every 3 months in our outpatient heart failure clinic.

Events of hospitalization, transplantation or death were assessed via the patient’s medical record or by telephone contact with patients, their relatives or general practitioner. Improvement of LVEF during follow-up was defined as ≥10% increase in LVEF or LVEF ≥ 50%. Progression to DCM was defined as >12 months persistence of heart failure symptoms and reduced LVEF and verified by CMR and EMB in individual cases [3,19].

### 2.5. Statistical Analysis

Categorical variables are reported as absolute and relative values, continuous variables as mean ± SEM or median (interquartile range, IQR) according to the normality of the distribution. For comparison of categorical data, the chi-square test or Fisher’s exact test was used. Continuous data were compared using the Student’s *t*-test or Mann–Whitney *U* test, as appropriate. The association between continuous and categorical data was assessed by multiple logistic regression. A *p*-Value of <0.05 was considered statistically significant. 

All analyses were performed with SigmaPlot 14.0 (Systat Software, San Jose, CA, USA) and R 3.6.0. (A Language and Environment for Statistical Computing, Vienna, Austria, 2019).

## 3. Results

### 3.1. Study Population

**Figure 1:**
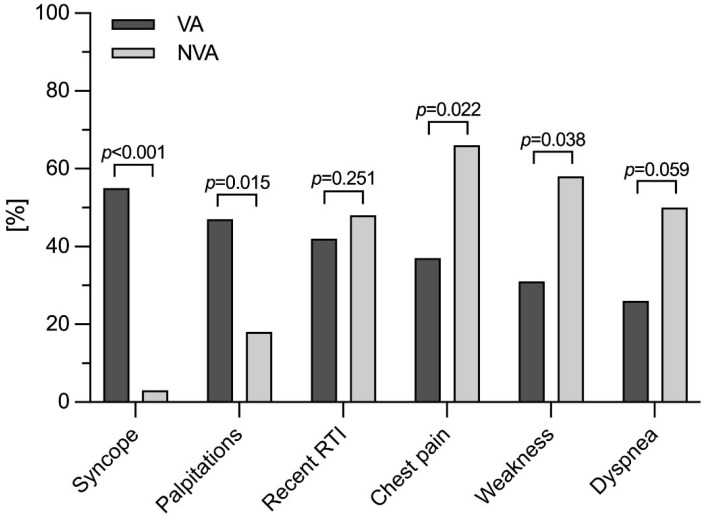
Clinical symptoms differ between VA vs. NVA patients. Symptoms at initial admission are presented for VA vs. NVA patients. NVA, non-ventricular arrhythmia; RTI, respiratory tract infection; VA, ventricular arrhythmia.

Detailed baseline characteristics of VA vs. NVA patients with myocarditis are shown in Table 1. Patients with VA more frequently experienced syncope (21/38, 55% vs. 1/38, 3%; *p* < 0.001) and palpitations (18/38, 47% vs. 7/38, 18%; *p* = 0.015) but less often reported chest pain (14/38, 37% vs. 25/38, 66%; *p* = 0.022) and weakness (12/38, 31% vs. 22/38, 58%; *p* = 0.038) (Figure 1). 

### 3.2. Ventricular Arrhythmia Characteristics

Most patients with a history of VA initially presented in sinus rhythm (35/38, 92%), with a mean heart rate of 69 ± 3 bpm. Bundle branch block or first-degree atrioventricular block could be detected in 7/38 (18%) patients, repolarization abnormalities including ST segment elevation or depression and T wave negativity in 17/38 (45%). Predominant VA was monomorphic ventricular tachycardia (VT) in 21/38 (55%) patients, followed by premature ventricular complexes (PVC) in 19/38 (50%) and ventricular fibrillation (VF) in 11/38 (29%) patients. PVC was mostly monomorphic, with a mean burden of 13 ± 2.5%, and the only documented VA in 10/38 (26%) patients. 

### 3.3. Diagnostic Characteristics

Mean C-reactive protein (25 ± 6 mg/L vs. 54 ± 12 mg/L; *p* = 0.003) and mean N-terminal prohormone of brain natriuretic peptide (3584 ± 1984 ng/L vs. 3959 ± 1099 ng/L; *p* = 0.040) were smaller in VA than in NVA patients. Median high-sensitivity cardiac troponin T did not differ between groups (54 (13.25–549.5) pg/mL vs. 102 (24.5–408.5) pg/mL; *p* = 0.568) (Table 1).

Echocardiographic studies at baseline revealed mildly reduced LVEF in both groups, with a mean LVEF of 47 ± 2.3% (VA) vs. 40 ± 3% (NVA) (*p* = 0.069). Systolic dysfunction of the right ventricle with a tricuspid annular plane systolic excursion of <17 mm was documented in 2/38 (5%) patients in the VA group and in 9/38 (24%) in the NVA group (*p* = 0.050). Coronary angiography was performed more often in VA patients (36/38, 95% vs. 26/38, 68%; *p* = 0.008) and revealed non-obstructive coronary artery disease in 7/36 (18%) and 3/26 (12%) patients, respectively (*p* = 0.499).

EMB to confirm diagnosis was conducted in 28/38 (74%) VA and 21/38 (55%) NVA patients (*p* = 0.150), with persistence of viral infection seen in 6/28 (21%) vs. 8/21 (38%), respectively (*p* = 0.585).

### 3.4. Cardiac Magnetic Resonance Imaging

**Figure 2:**
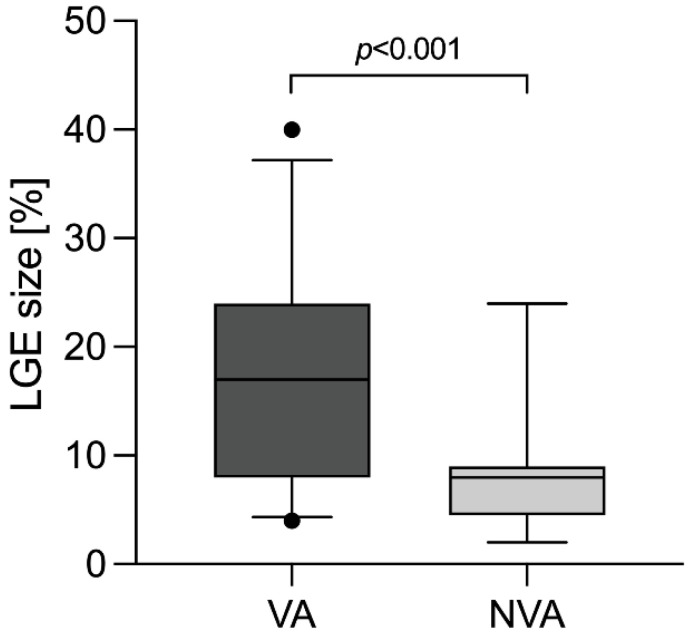
LGE size in % of left ventricular myocardial mass. LGE, late gadolinium enhancement; NVA, non-ventricular arrhythmia; VA, ventricular arrhythmia.

CMR was performed in 34/38 (90%) VA and 35/38 (92%) NVA patients. LGE and myocardial edema were found in 32/34 (94%) vs. 24/35 (69%) (*p* = 0.016) and 17/34 (50%) vs. 9/35 (26%) (*p* = 0.067) VA and NVA patients, respectively. Further retrospective LGE quantification could be conducted in 26/32 (81%) VA and 17/24 (71%) NVA patients (*p* = 0.553). Mean LGE extent was larger in VA than in NVA patients, incorporating 17.6 ± 1.8% vs. 8.2 ± 1.3% of left ventricular myocardial mass, respectively (*p* < 0.001) (Figure 2). Similarly, LGE extent according to the 17-segment model of the American Heart Association consisted of 5.5 ± 0.5 vs. 3.9 ± 0.5 segments (*p* = 0.062). The most common localization of LGE in the VA group was mid-inferolateral (16/26 patients, 62%), followed by mid-anterolateral (14/26 patients, 54%), mid-inferior and basal-inferolateral (11/26 patients each, 42%). In the NVA group, the most common localization of LGE was basal-inferolateral (9/17 patients, 53%), mid-inferolateral (8/17 patients, 47%) and basal-anterolateral (7/17 patients, 41%). Detailed CMR characteristics are listed in Table 2. 

### 3.5. Myocarditis Treatment

There was no difference in immunosuppressive therapy, initiated in 9/38 VA (24%) and 8/38 NVA (21%) patients (*p* = 0.952) (Table 1). Upon initial admission, only patients with a history of VA required hemodynamic support, either by extracorporeal membrane oxygenation in three patients or percutaneously inserted ventricular assist device (Impella^®^, Abiomed Inc., Danvers, MA, USA) in one patient. 

### 3.6. Follow-Up

Follow-up characteristics are shown in Table 3. During follow-up, 14/36 (39%) VA patients experienced recurrent VA. There was no difference in initial LVEF between patients with and without recurrent VA (49 ± 3% vs. 45 ± 3%; *p* = 0.509). Reduced LVEF at baseline and during follow-up did not predict recurrent VA (OR 1.02 (95% CI 0.97–1.07), *p* = 0.498). 

Invasive electrophysiological study was performed in 20/38 (53%) VA patients 6.6 ± 1.7 months after the diagnosis of myocarditis. Of these, 12 received right ventricular stimulation for arrhythmic risk stratification and guidance of therapeutic decision making, with or without induction of sustained VT (6/12 each, 50%). Of the inducible patients, 3/6 (50%) patients underwent RFCA during further follow-up. WCD prescription with and without following ICD implantation was conducted in 1/6 (17%) patients each, ICD implantation alone in 3/6 (50%). RFCA for VA was initially conducted in 9/20 (45%) patients— 2 for VT and 7 for PVC—all targeted in the left ventricle. PVC were mainly located in the postero-basal/-septal/-lateral left ventricle (4/7, 57%). Other localizations were the left ventricular outflow tract, the superior mitral valve annulus, the left coronary cusp and the great cardiac vein. Altogether, the origin of clinically observed VT and PVC correlated with the location of myocardial scar and LGE detected in previously conducted CMR.

During a follow-up of 24 ± 3 months, 5/9 (56%) patients remained free from any sustained VA. Comparing patients with immunosuppressive therapy (9/38, 24%) vs. those without (29/38, 76%), initial RFCA was performed in 3/9 (33%) vs. 6/29 (21%) patients (*p* = 0.655). Of those, 3/3 (100%) vs. 2/6 (33%) experienced VA recurrence during follow-up (*p* = 0.167). 

**Figure 3:**
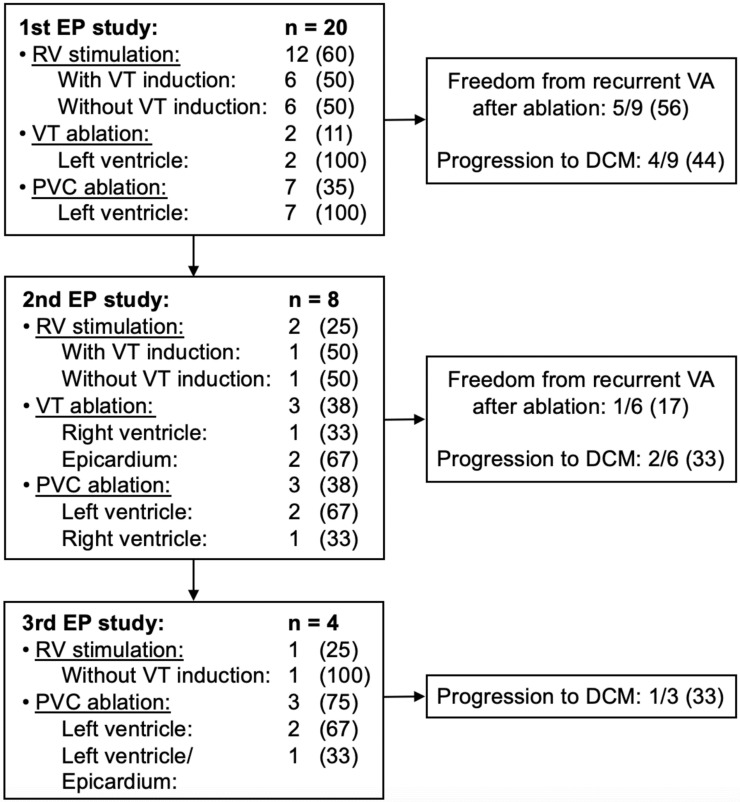
Ablation targets and outcome of myocarditis patients with recurrent VA. Values are indicated as n (%). DCM, dilated cardiomyopathy; EP, electrophysiological; PVC, premature ventricular complexes; RV, right ventricular; VA, ventricular arrhythmia; VT, ventricular tachycardia.

Recurrent electrophysiological study was performed in 8/20 patients 4.6 ± 1.2 after the first one and included right ventricular stimulation with or without VT induction (one patient, each), right ventricular (one patient) and epicardial VT (two patients) and left ventricular PVC ablation (three patients). A third electrophysiological study was conducted in four patients, with one receiving endo-epicardial PVC ablation after previous right ventricular stimulation with VT induction and epicardial VT ablation (Figure 3). 

**Figure 4:**
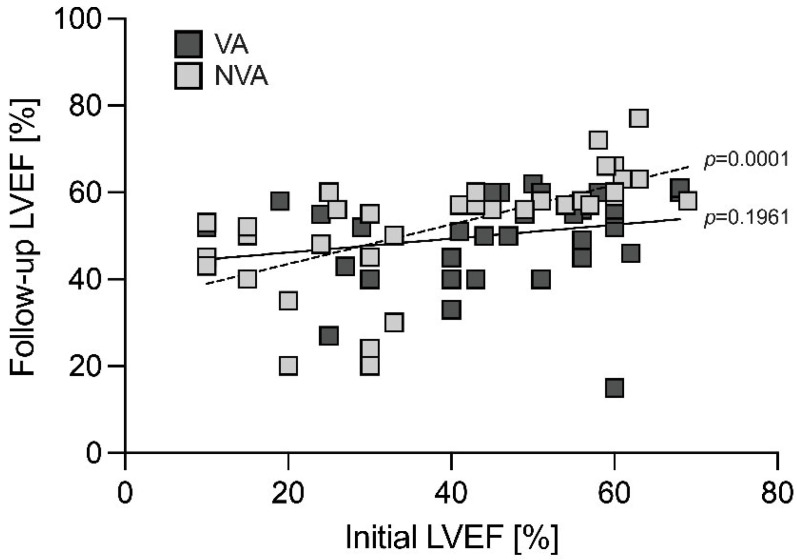
LVEF at baseline and during follow-up is illustrated for VA vs. NVA patients. Whereas the dashed line demonstrates a correlation between LVEF at baseline and during follow-up in the NVA group, there is no correlation in the VA group (solid line). LVEF, left ventricular ejection fraction; NVA, non-ventricular arrhythmia; VA, ventricular arrhythmia.

LVEF at baseline and during follow-up correlated in NVA patients (*p* = 0.0001), but not in those presenting with VA (*p* = 0.1961) (Figure 4). ICD implantation was conducted more often in VA vs. NVA patients (47% vs. 17%; *p* = 0.014). 

Disease progression to DCM with LVEF ≤ 45% was seen in 10/36 (28%) VA and 9/35 (26%) NVA patients (*p* = 1.0), who initially presented with a LVEF of 45 ± 4% vs. 21 ± 3% (*p* < 0.001) (Table 3). Among VA patients, there was no difference in baseline LVEF between those with vs. without disease progression to DCM (45 ± 4% vs. 47 ± 5%; *p* = 0.446). Among NVA patients, baseline LVEF was lower in those with persistent or progressive heart failure (21 ± 2.6% vs. 46 ± 3%; *p* < 0.001).

One VA patient and two NVA patients with severe heart failure were listed for transplantation. Three VA patients (LVEF 50 ± 8%) and one NVA (LVEF 30%) patient died 5.6 ± 3 and 28 months after the initial diagnosis, respectively.

### 3.7. Cases with Fatal Outcome

Firstly, one 34-year-old male patient with initial VF and myocarditis refused ICD implantation. LVEF was preserved at baseline and after 3 months wearing a WCD. Recordings documented PVC, but no sustained VA. Follow-up CMR depicted persistent LGE without evidence of acute inflammation. Still refusing ICD implantation, the patient was re-admitted to hospital several months later due to recurrent VT and VF. He was resuscitated multiple times and died despite escalated intensive care.

An 18-year-old woman was admitted to hospital due to VT and PVC. At presentation, LVEF was severely impaired (30%), and CMR showed both LGE (16% of left ventricular myocardial mass) and myocardial edema as a correlate of acute myocarditis. EMB revealed chronic lymphocytic myocarditis, and immunosuppressive therapy with prednisone and azathioprine was established for 5 months. Two months after receiving a WCD, echocardiography depicted normalized LVEF. Six months after initial diagnosis, the patient collapsed; bystander resuscitation was started after 5 minutes and, later on, brain death was diagnosed.

Lastly, a 26-year-old woman was found unconscious and cyanotic at home. After cardiopulmonary resuscitation by an emergency physician, return of spontaneous circulation with initially mildly impaired LVEF could be established. In the course, the patient died due to cardiogenic shock, with post-mortem biopsy revealing the initial diagnosis of subacute myocarditis.

In the NVA group, a 31-year-old male patient died 15 months after heart transplantation due to septic shock.

## 4. Discussion

The main findings of the present study investigating characteristics and prognostic relevance of VA in patients with myocarditis are as follows: (1) Reduced LVEF upon initial presentation and during follow-up does not predict VA in patients with myocarditis; (2) patients with VA are more likely to show LGE in CMR, with a larger LGE extent than those without VA; (3) RFCA, guided by pre-procedural imaging, should be considered for treatment of VA in patients with myocarditis.

### 4.1. Prognostic Value of Left Ventricular Ejection Fraction

Impaired LVEF is associated with VA and sudden cardiac death and has been described as a predictor of poor long-term outcome in patients with active myocarditis [3,5], but so far, the prognostic value of LVEF in patients with myocarditis and VA is unknown.

In the presented study, reduced LVEF predicted occurrence of VA neither at admission nor during follow-up and was further not associated with progression to DCM in VA patients. Interestingly, among the three VA patients who died during follow-up, mean LVEF at baseline was 47 ± 12% and did not deteriorate but actually improved. This observation might call into question the relevance of LVEF as the most important prognostic parameter [3] in patients with myocarditis and VA, illustrating the need for prospective multi-center studies on the management and outcome in this population.

### 4.2. Importance of Cardiac Magnetic Resonance Imaging

The value of CMR in patients with suspected myocarditis has substantially increased during the last decade, making it an essential part for confirmation of diagnosis nowadays [5]. Nevertheless, there are still limited prognostic CMR data in myocarditis [20].

Presence of LGE has been described as the best independent predictor of all-cause mortality and cardiac mortality, including survived sudden cardiac death due to ICD discharge. Remarkably, in a case series of patients with biopsy-proven viral myocarditis, those without LGE did not experience sudden cardiac death during follow-up, even if LVEF was severely impaired [21]. Classification of patients with suspected acute myocarditis into different groups according to the main clinical pattern of presentation has demonstrated various extents of LGE, with patients presenting with arrhythmias or heart failure displaying a larger number of left ventricular segments with LGE than those with an infarct-like pattern [22]. These results are in line with the here-presented study, illustrating that patients with VA are more likely to show LGE in CMR, incorporating a greater part of left ventricular mass than those without VA. The high LGE incidence of 94% in patients with VA might consequently suggest a prognostic value of VA in the long-term.

However, LGE is not always present in patients with myocarditis. CMR sensitivity has been found to correlate with the extent of cell necrosis promoting the expansion of interstitial space and to be lower in patients with an arrhythmic pattern than in those with a cardiomyopathic or an infarct-like pattern [23]. This observation might be explained by a lower diagnostic imaging quality due to recurrent arrhythmia in these patients [23,24]. Furthermore, consideration of different disease stages, with patients with both acute and chronic myocarditis included in the here presented study, might have affected the incidence of LGE in CMR compared to prior results.

Whereas inferolateral segments have previously been found to be the most frequently involved ones in all subgroups of myocarditis patients, mid-layer left ventricular LGE was more likely to be detected among myocarditis patients presenting with arrhythmias or heart failure [22]. Similarly, in our study, the most common localization of LGE was inferolateral in both groups, followed by varying other localizations. Whether these different LGE localizations have a prognostic impact in patients with myocarditis or contribute to the development of VA needs to be determined.

### 4.3. Treatment of Ventricular Arrhythmia in Patients with Myocarditis

Myocarditis may provoke the occurrence of VA both in its acute and chronic stage by inflammatory infiltration and cell necrosis or immune reaction, fibrosis and resulting electric remodeling, respectively [11]. According to the current guidelines, antiarrhythmic drug therapy should be firstly considered during the acute phase, deferring ICD implantation until resolution [5].

**Figure 5:**
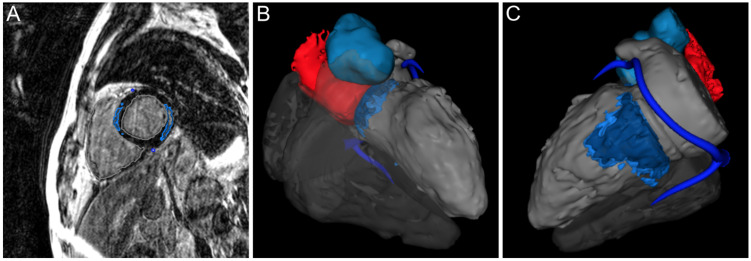
Pre-procedural imaging guides catheter ablation in patients with myocarditis. Exemplary case of a patient with myocarditis who presented with symptomatic premature ventricular complexes (inferior axis, positive in lead I) and non-sustained ventricular tachycardia (CL 258 ms). The patient underwent CMR for 3D imaging acquisition before catheter ablation. (**A**) Short-axis view of CMR depicting dense scar in a septal and lateral area. (**B**,**C**) 3D reconstruction of CMR guides intra-procedural electroanatomic mapping and catheter ablation. Mapping in the right ventricular outflow tract demonstrated earliest activation (42 ms) in the area of septal dense scar and ablation at this localization resulted in complete suppression of premature ventricular complexes and non-sustained ventricular tachycardia. CMR, cardiac magnetic resonance imaging.

However, especially the treatment of drug-refractory VA or recurrent electrical storm may be challenging [25]. Even though RFCA has been sporadically reported for patients with myocarditis and recurrent VA, specific recommendations do not exist [5]. In a consecutive series of patients with chronic active myocarditis, RFCA for drug-refractory VT has been proven to be safe and effective with an acute success rate of 70% after endocardial RFCA and 90% freedom from recurrent VT during follow-up after additional epicardial RFCA [11]. Considering the high incidence of subepicardial and mid-wall scar extension, a first line endo-epicardial ablation approach, based on pre-procedural imaging, has further been suggested in order to adequately target the underlying substrate, which therefore might improve long-term success rates in some patients [26,27]. As underlined by the correlation between VA origin and location of myocardial scar and LGE in the here presented study, pre-procedural 3D reconstruction of CMR imaging is gaining an increasing role for illustration of scar areas guiding RFCA in this population (Figure 5). 

In line with these observations, our findings now confirm the feasibility and potential advantage of RFCA in patients with myocarditis, reinforcing the necessity of an oftentimes additional epicardial approach. However, the timing of RFCA remains challenging. Recently, after first-line endo-epicardial ablation 20% of myocarditis patients have been found with recurrent VT during a follow-up of 12 months, proving ongoing active inflammation, verified by EMB or second-level imaging, as the only predictor of VT recurrence [28]. Consequently, evaluation of the best timing of RFCA, as well as the optimal candidates for RFCA considering the often heterogenous symptoms during acute inflammation, is needed.

## 5. Conclusions

Impaired LVEF in patients with myocarditis does not predict occurrence of VA at initial admission and during follow-up. Patients with VA are more likely to show LGE in CMR, with a larger LGE extent of myocardial mass than those without VA. RFCA, guided by pre-procedural imaging, should be considered for treatment of recurrent VA in patients with myocarditis.

## Figures and Tables

**Table 1:** Patient baseline characteristics.

Variable	VA (n = 38)	NVA (n = 38)	*p* Value
Age	45 ± 3	38 ± 2	0.074
Male sex	26 (68)	32 (84)	0.109
Symptoms			
Syncope	21 (55)	1 (3)	<0.001
Chest pain	14 (37)	25 (66)	0.022
Dyspnea	10 (26)	19 (50)	0.059
Weakness	12 (31)	22 (58)	0.038
Recent respiratory infection	16 (42)	22 (58)	0.251
Palpitations	18 (47)	7 (18)	0.015
Laboratory			
Hs-cTnT, pg/mL	54 (13.15–549.5)	102 (23.5–408.5)	0.568
*Hs-cTnT < 14 pg/mL*	*8 (25)*	*3 (9)*	*0.168*
CRP, mg/L	25 ± 6	54 ± 12	0.003
*CRP < 5 mg/L*	*18 (53)*	*6 (17)*	*0.004*
NT-proBNP, ng/L	3584 ± 1984	3959 ± 1099	0.040
*NT-proBNP < 248 ng/L*	*9 (32)*	*5 (18)*	*0.314*
Echocardiography			
LVEF, %	47 ± 2	40 ± 3	0.069
LVEDD, mm	54 ± 2	53 ± 2	0.956
LVEF < 30%	6 (16)	12 (32)	0.177
LVEF ≥ 54%	16 (42)	12 (32)	0.476
TAPSE < 17 mm	2 (5)	9 (13)	0.050
Endomyocardial biopsy			
Positive EMB	22 (79)	20 (95)	0.214
*Active myocarditis*	*12 (55)*	*12 (60)*	*0.964*
*Borderline myocarditis*	*6 (27)*	*5 (25)*	*0.854*
*Previous myocarditis*	*4 (18)*	*3 (15)*	*1.0*
Detection of viral infection	6 (27)	8 (40)	0.585
*Parvovirus B19*	*2 (33)*	*6 (75)*	*0.277*
*Human herpesvirus 6*	*4 (66)*	*1 (13)*	*0.091*
*Epstein-Barr virus*	*0 (0)*	*1 (13)*	*1.0*
Therapy			
Immunosuppression	9 (24)	8 (21)	0.952
*Azathioprine + Prednisolone*	*5 (56)*	*7 (88)*	*0.294*
*Cyclosporine + MMF + Prednisolone*	*1 (11)*	*0 (0)*	*1.0*
*Cyclosporine + Prednisolone*	*0 (0)*	*1 (13)*	*1.0*
*Azathioprine* *Interferone* *Prednisolone*	*1 (11)* *1 (11)* *1 (11)*	*0 (0)* *0 (0)* *0 (0)*	*1.0* *1.0* *1.0*
Beta-receptor blockers	31 (82)	25 (66)	0.192
Amiodarone	7 (18)	1 (3)	0.056

Data are presented as mean ± SEM, median (IQR) or n (%). CRP, C-reactive protein; EMB, endomyocardial biopsy; Hs-cTnT, high-sensitivity cardiac troponin T; MMF, mycophenolate mofetil; NT-proBNP, N-terminal prohormone of brain natriuretic peptide; NVA, non-ventricular arrhythmia; LVEDD, left ventricular end-diastolic diameter; LVEF, left ventricular ejection fraction; TAPSE, tricuspid annular plane systolic excursion; VA, ventricular arrhythmia.

**Table 2:** Cardiac magnetic resonance imaging characteristics.

Variable	VA (n = 34)	NVA (n = 35)	*p*-Value
LGE	32 (94)	24 (69)	0.016
Edema	17 (50)	9 (26)	0.067
LGE mass, %	17.6 ± 1.8	8.2 ± 1.3	<0.001
LGE mass, g	24.7 ± 3.2	11.8 ± 2.4	0.002
Segments with LGE	5.5 ± 0.5	3.9 ± 0.5	0.062
LVEDV, mL	179.6 ± 12.1	180.7 ± 10.8	0.849
LVESV, mL	90 ± 9.5	102.8 ± 12	0.693
LVSV, mL	90 ± 5.1	80.9 ± 5.8	0.210
LVEF, %	52.3 ± 2.2	47.2 ± 3.4	0.595
LVEDM, g	156.1 ± 7.1	159 ± 7.1	0.665

Data are presented as mean ± SEM or n (%). LGE, late gadolinium enhancement; LVEDM, left ventricular end-diastolic myocardial mass; LVEDV, left ventricular end-diastolic volume, LVEF, left ventricular ejection fraction; LVESV, left ventricular end-systolic volume; LVSV, left ventricular systolic volume; NVA, non-ventricular arrhythmia; VA, ventricular arrhythmia.

**Table 3:** Follow-up characteristics.

Variable	VA (n = 36)	NVA (n = 35)	*p* Value
LVEF, %	51 ± 2	53 ± 2	0.326
DCM with LVEF ≤ 45%	10 (28)	9 (26)	1.0
*Initial LVEF, %*	*45 ± 4*	*21 ± 3*	*<0.001*
WCD equipment	14 (39)	6 (17)	0.076
*ICD over time*	*5 (36)*	*1 (17)*	*0.613*
LVEF improvement	12 (33)	18 (52)	1.0
ICD implantation	17 (47)	6 (17)	0.014
*ICD shock delivery*	*5 (29)*	*1 (17)*	*1.0*

Data are presented as mean ± SEM or n (%). DCM, dilated cardiomyopathy; ICD, implantable cardioverter-defibrillator; LVEF, left ventricular ejection fraction; NVA, non-ventricular arrhythmia; VA, ventricular arrhythmia; WCD, wearable cardioverter-defibrillator.

## Data Availability

The data underlying this article will be shared upon reasonable request to the corresponding author.

## References

[1] Tung R., Bauer B., Schelbert H., Lynch J.P., Auerbach M., Gupta P., Schiepers C., Chan S., Ferris J., Barrio M. (2015). Incidence of abnormal positron emission tomography in patients with unexplained cardiomyopathy and ventricular arrhythmias: The potential role of occult inflammation in arrhythmogenesis. Heart Rhythm.

[2] Lakkireddy D., Turagam M.K., Yarlagadda B., Dar T., Hamblin M., Krause M., Parikh V., Bommana S., Atkins D., Di Biase L. (2019). Myocarditis Causing Premature Ventricular Contractions: Insights from the MAVERIC Registry. Circ. Arrhythm. Electrophysiol..

[3] Anzini M., Merlo M., Sabbadini G., Barbati G., Finocchiaro G., Pinamonti B., Salvi A., Perkan A., Di Lenarda A., Bussani R. (2013). Long-term evolution and prognostic stratification of biopsy-proven active myocarditis. Circulation.

[4] Caforio A.L., Pankuweit S., Arbustini E., Basso C., Gimeno-Blanes J., Felix S.B., Fu M., Heliö T., Heymans S., Jahns R. (2013). Current state of knowledge on aetiology, diagnosis, management, and therapy of myocarditis: A position statement of the European Society of Cardiology Working Group on Myocardial and Pericardial Diseases. Eur. Heart. J..

[5] Priori S.G., Blomström-Lundqvist C., Mazzanti A., Blom N., Borggrefe M., Camm J., Elliott P.M., Fitzsimons D., Hatala R., Hindricks G. (2015). 2015 ESC Guidelines for the management of patients with ventricular arrhythmias and the prevention of sudden cardiac death: The Task Force for the Management of Patients with Ventricular Arrhythmias and the Prevention of Sudden Cardiac Death of the European Society of Cardiology (ESC). Endorsed by: Association for European Paediatric and Congenital Cardiology (AEPC). Eur. Heart. J..

[6] Te A.L.D., Wu T.C., Lin Y.J., Chen Y.Y., Chung F.P., Chang S.L., Lo L.W., Hu Y.F., Tuan T.C., Chao T.F. (2017). Increased risk of ventricular tachycardia and cardiovascular death in patients with myocarditis during the long-term follow-up: A national representative cohort from the National Health Insurance Research Database. Medicine.

[7] Richardson P., McKenna W., Bristow M., Maisch B., Mautner B., O’Connell J., Olsen E., Thiene G., Goodwin J., Gyarfas I. (1996). Report of the 1995 World Health Organization/International Society and Federation of Cardiology Task Force on the Definition and Classification of cardiomyopathies. Circulation.

[8] Aretz H.T., Billingham M.E., Edwards W.D., Factor S.M., Fallon J.T., Fenoglio J.J., Olsen E.G., Schoen F.J. (1987). Myocarditis. A histopathologic definition and classification. Am. J. Cardiovasc. Pathol..

[9] Caforio A.L., Calabrese F., Angelini A., Tona F., Vinci A., Bottaro S., Ramondo A., Carturan E., Iliceta S., Thiene G. (2007). A prospective study of biopsy-proven myocarditis: Prognostic relevance of clinical and aetiopathogenetic features at diagnosis. Eur. Heart. J..

[10] Narducci M.L., Rio T., Perna F., D’Amario D., Merlino B., Marano R., Bencardino G., Inzani F., Pelargonio G., Crea F. (2014). A Challenging Case Of Ventricular Arrhythmia In A Patient With Myocarditis: ICD Yes/No After Ablation. J. Atr. Fibrillation..

[11] Dello Russo A., Casella M., Pieroni M., Pelargonio G., Bartoletti S., Santangeli P., Zucchetti M., Innocenti E., Di Biase L., Carbucicchio C. (2012). Drug-refractory ventricular tachycardias after myocarditis: Endocardial and epicardial radiofrequency catheter ablation. Circ. Arrhythm. Electrophysiol..

[12] Schwarzl J.M., Schleberger R., Kahle A.K., Höller A., Schwarzl M., Schaeffer B.N., Münkler P., Moser J., Akbulak R.Ö., Eickholt C. (2021). Specific electrogram characteristics impact substrate ablation target area in patients with scar-related ventricular tachycardia-insights from automated ultrahigh-density mapping. J. Cardiovasc. Electrophysiol..

[13] Friedrich M.G., Sechtem U., Schulz-Menger J., Holmvang G., Alakija P., Cooper L.T., White J.A., Abdel-Aty H., Gutberlet M., Prasad S. (2009). Cardiovascular magnetic resonance in myocarditis: A JACC White Paper. J. Am. Coll. Cardiol..

[14] Radunski U.K., Lund G.K., Säring D., Bohnen S., Stehning C., Schnackenburg B., Avanesov M., Tahir E., Adam G., Blankenberg S. (2017). T1 and T2 mapping cardiovascular magnetic resonance imaging techniques reveal unapparent myocardial injury in patients with myocarditis. Clin. Res. Cardiol..

[15] Radunski U.K., Lund G.K., Stehning C., Schnackenburg B., Bohnen S., Adam G., Blankenberg S., Muellerleile K. (2014). CMR in patients with severe myocarditis: Diagnostic value of quantitative tissue markers including extracellular volume imaging. J. Am. Coll. Cardiol. Img..

[16] Schulz-Menger J., Bluemke D.A., Bremerich J., Flamm S.D., Fogel M.A., Friedrich M.G., Kim R.J., von Knobelsdorff-Brenkenhoff F., Kramer C.M., Pennell D.J. (2020). Standardized image interpretation and post-processing in cardiovascular magnetic resonance—2020 update: Society for Cardiovascular Magnetic Resonance (SCMR): Board of Trustees Task Force on Standardized Post-Processing. J. Cardiovasc. Magn. Reson..

[17] Cerqueira M.D., Weissman N.J., Dilsizian V., Jacobs A.K., Kaul S., Laskey W.K., Pennell D.J., Rumberger J.A., Ryan T., Verani M.S. (2002). Standardized myocardial segmentation and nomenclature for tomographic imaging of the heart. A statement for healthcare professionals from the Cardiac Imaging Committee of the Council on Clinical Cardiology of the American Heart Association. Circulation.

[18] McDonagh T.A., Metra M., Adamo M., Gardner R.S., Baumbach A., Böhm M., Burri H., Butler J., Celutkiene J., Chioncel O. (2021). 2021 ESC Guidelines for the diagnosis and treatment of acute and chronic heart failure: Developed by the Task Force for the diagnosis and treatment of acute and chronic heart failure of the European Society of Cardiology (ESC) With the special contribution of the Heart Failure Association (HFA) of the ESC. Eur. Heart. J..

[19] Ghimire A., Fine N., Ezekowitz J.A., Howlett J., Youngson E., McAlister F.A. (2019). Frequency, predictors, and prognosis of ejection fraction improvement in heart failure: An echocardiogram-based registry study. Eur. Heart. J..

[20] Vermes E., Childs H., Faris P., Friedrich M.G. (2014). Predictive value of CMR criteria for LV functional improvement in patients with acute myocarditis. Eur. Heart. J. Cardiovasc. Imaging..

[21] Grün S., Schumm J., Greulich S., Wagner A., Schneider S., Bruder O., Kispert E.M., Hill S., Ong P., Klingel K. (2012). Long-term follow-up of biopsy-proven viral myocarditis: Predictors of mortality and incomplete recovery. J. Am. Coll. Cardiol..

[22] Di Bella G., Camastra G., Monti L., Dellegrottaglie S., Piaggi P., Moro C., Pepe A., Lanzillo C., Pontone G., Perazzolo Marra M. (2017). Left and right ventricular morphology, function and late gadolinium enhancement extent and localization change with different clinical presentation of acute myocarditis Data from the ITAlian multicenter study on MYocarditis (ITAMY). J. Cardiovasc. Med..

[23] Francone M., Chimenti C., Galea N., Scopelliti F., Verardo R., Galea R., Carbone I., Catalano C., Fedele F., Frustaci A. (2014). CMR sensitivity varies with clinical presentation and extent of cell necrosis in biopsy-proven acute myocarditis. J. Am. Coll. Cardiol. Img..

[24] Frustaci A., Chimenti C. (2007). Images in cardiovascular medicine. Cryptogenic ventricular arrhythmias and sudden death by Fabry disease: Prominent infiltration of cardiac conduction tissue. Circulation.

[25] Münkler P., Klatt N., Willems S., Meyer C. (2019). High-density mapping-based ablation strategy in a 30-year-old patient with a history of myocarditis. Europace.

[26] De Cobelli F., Pieroni M., Esposito A., Chimenti C., Belloni E., Mellone R., Canu T., Perseghin G., Faudio C., Maseri A. (2006). Delayed gadolinium-enhanced cardiac magnetic resonance in patients with chronic myocarditis presenting with heart failure or recurrent arrhythmias. J. Am. Coll. Cardiol..

[27] Maccabelli G., Tsiachris D., Silberbauer J., Esposito A., Bisceglia C., Baratto F., Colantoni C., Trecisi B., Palmisano A., Vergara P. (2014). Imaging and epicardial substrate ablation of ventricular tachycardia in patients late after myocarditis. Europace.

[28] Peretto G., Sala S., Basso C., Rizzo S., Radinovic A., Frontera A., Rosaio Limite L., Paglino G., Bisceglia C., De Luca A. (2020). Inflammation as a Predictor of Recurrent Ventricular Tachycardia After Ablation in Patients with Myocarditis. J. Am. Coll. Cardiol..

